# Supplementary figures and images for: Diffusion-based neuromodulation can eliminate catastrophic forgetting in simple neural networks
Source: PLoS One. 2017 Nov 16;12(11):e0187736. doi: 10.1371/journal.pone.0187736 (PMC5690421; doi:10.1371/journal.pone.0187736)

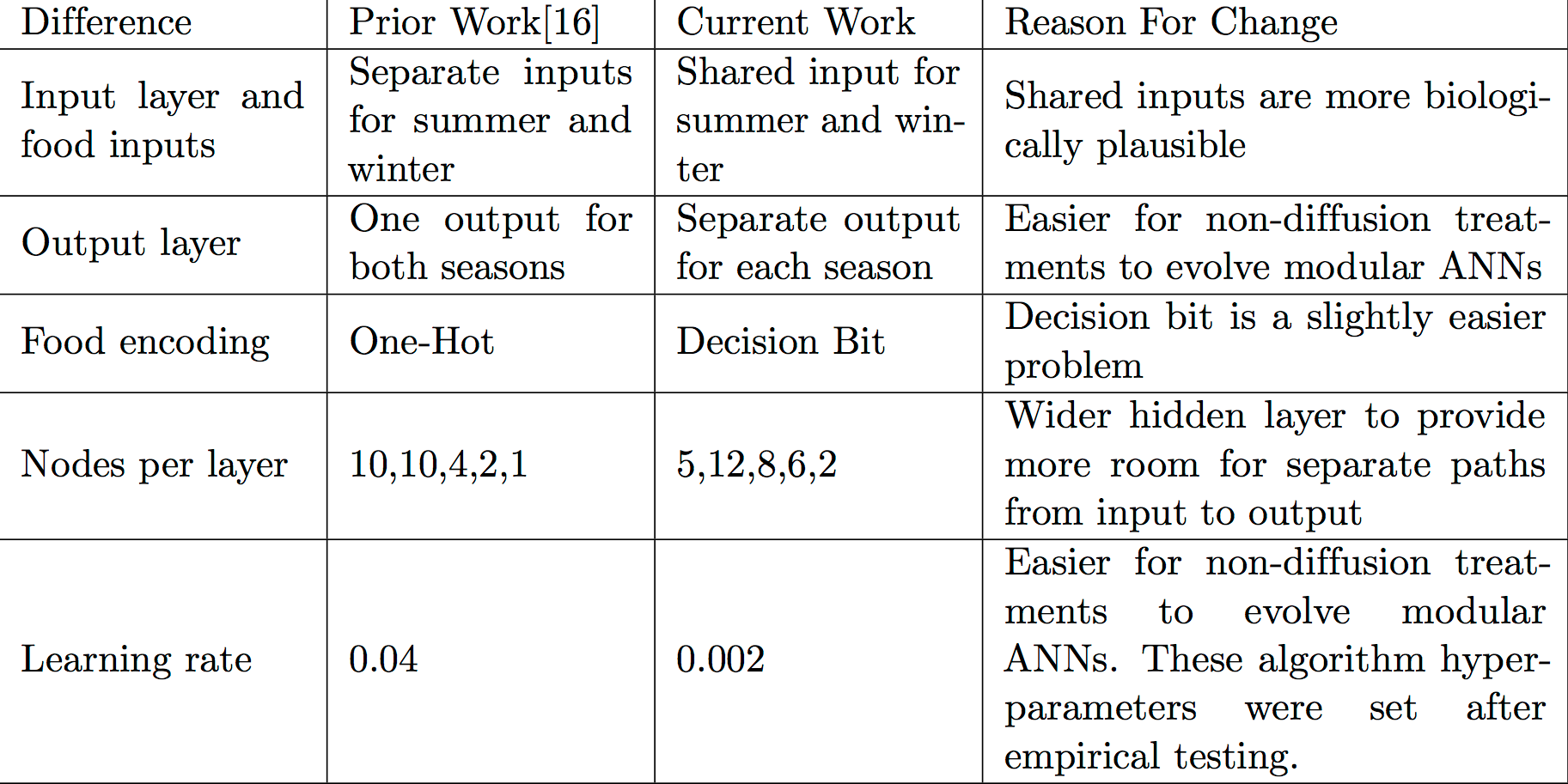

Supplement: S1 Table — Differences prevent direct comparison between the non-diffusion treatments in this work and the networks in Ellefsen et al. [16]. Purpose of many of the changes were to make it easier for modular solutions to appear in order to investigate whether they aid with catastrophic forgetting. (TIFF) [file pone.0187736.s001.tiff]

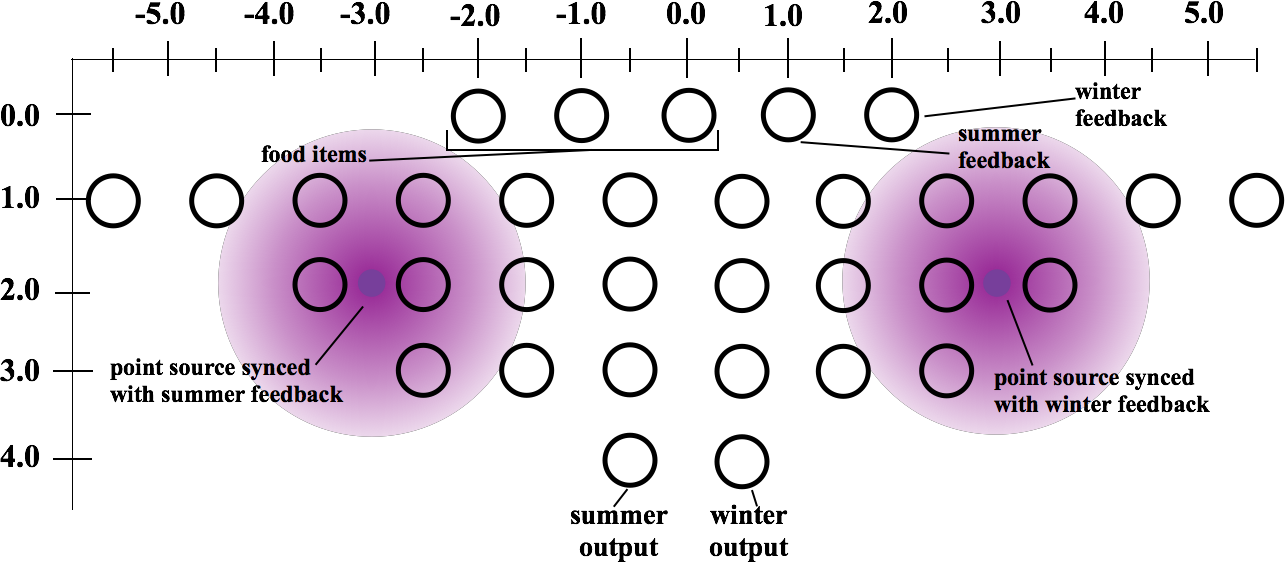

Supplement: S1 Fig — Individuals in the foraging task are represented as ANNs where each node possesses an (x, y) position. The first three inputs correspond to food items while the last two inputs are fed positive (1) and negative (−1) feedback signals for the summer and winter season respectively. An output greater than 0 results in the agent eating the food item presented. Two point sources, one for each season, exist at the locations (−3, 2), and (3, 2). Their activation is synchronized to the positive and negative feedback of the summer and winter season. They affect all nodes within a radius of 1.5 and the modulatory signal of the point sources increases as a Gaussian as you get closer (Eq 7). (TIF) [file pone.0187736.s002.tif]

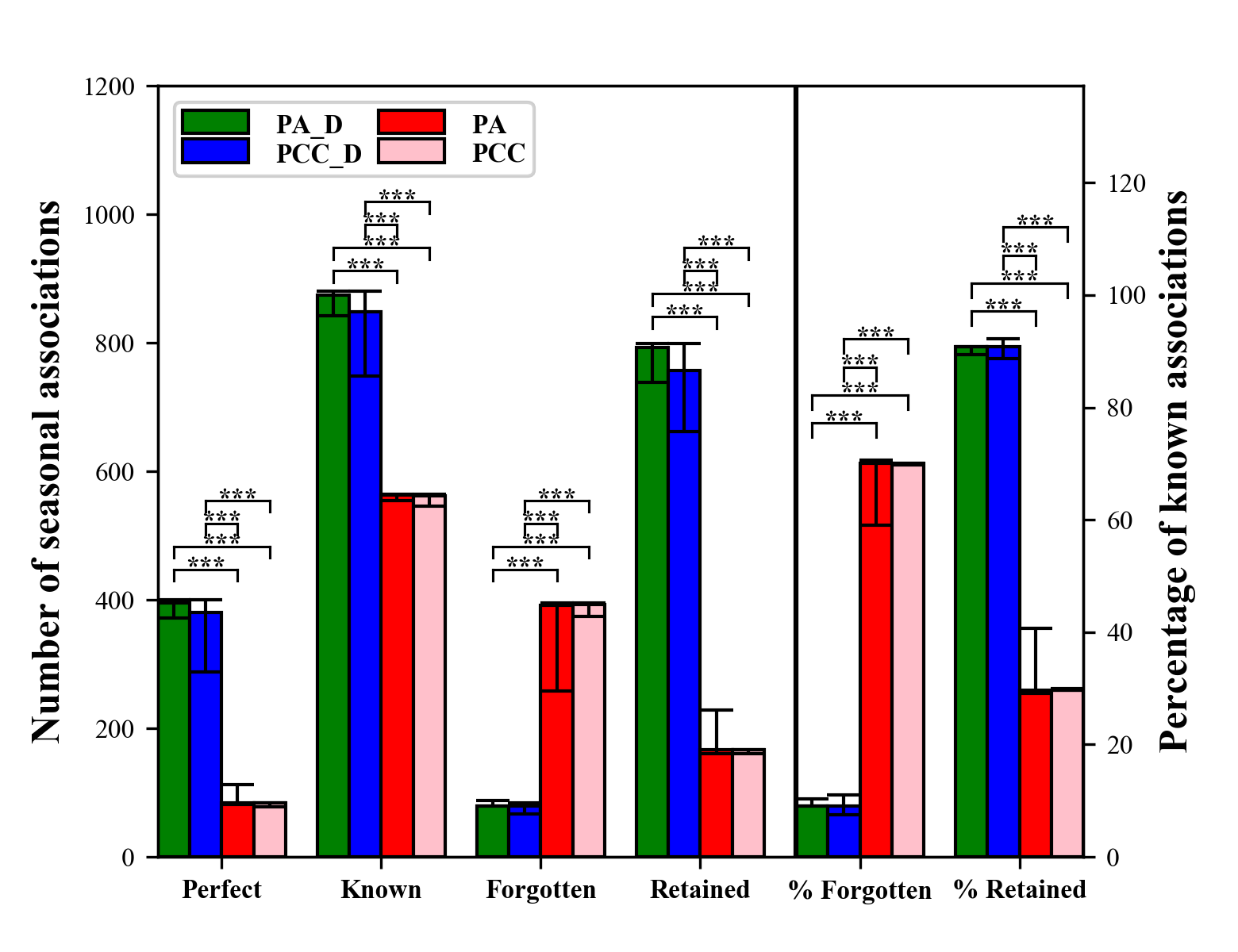

Supplement: S2 Fig — See main text for description and interpretation. For further details on seasonal associations see Ellefsen et al. [16]. (TIF) [file pone.0187736.s003.tif]

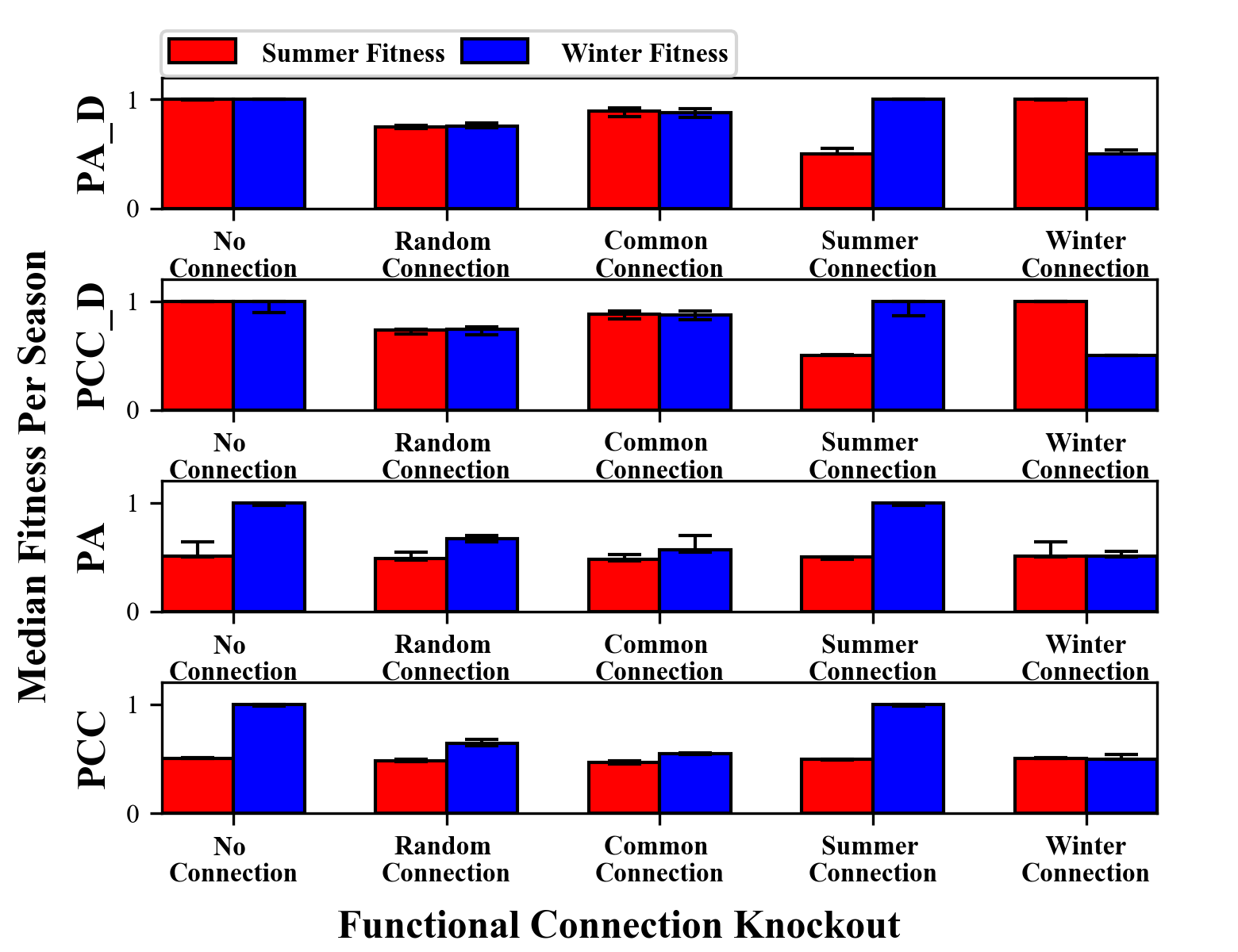

Supplement: S3 Fig — The original summer and winter fitness for all CFNs is plotted along with the summer and winter fitness after the knockout of a random, common, winter, or summer functional connection. The original (no connection) and random connection fitnesses are provided for comparison. For all treatments, the removal of a summer (or winter) functional connection only causes a drop in summer (or winter) fitness. In contrast, the removal of a common functional connection causes a drop in fitness for both seasons. For non-diffusion treatments, the drop in summer fitness is difficult to see because non-diffusion treatments do not have much competency (i.e. original fitness) on the summer task to begin with. The knockout analysis confirms that the summer and winter functional modules identified by ARK encode for those seasons respectively and that the common functional module identified by ARK encodes for both. (TIF) [file pone.0187736.s004.tif]

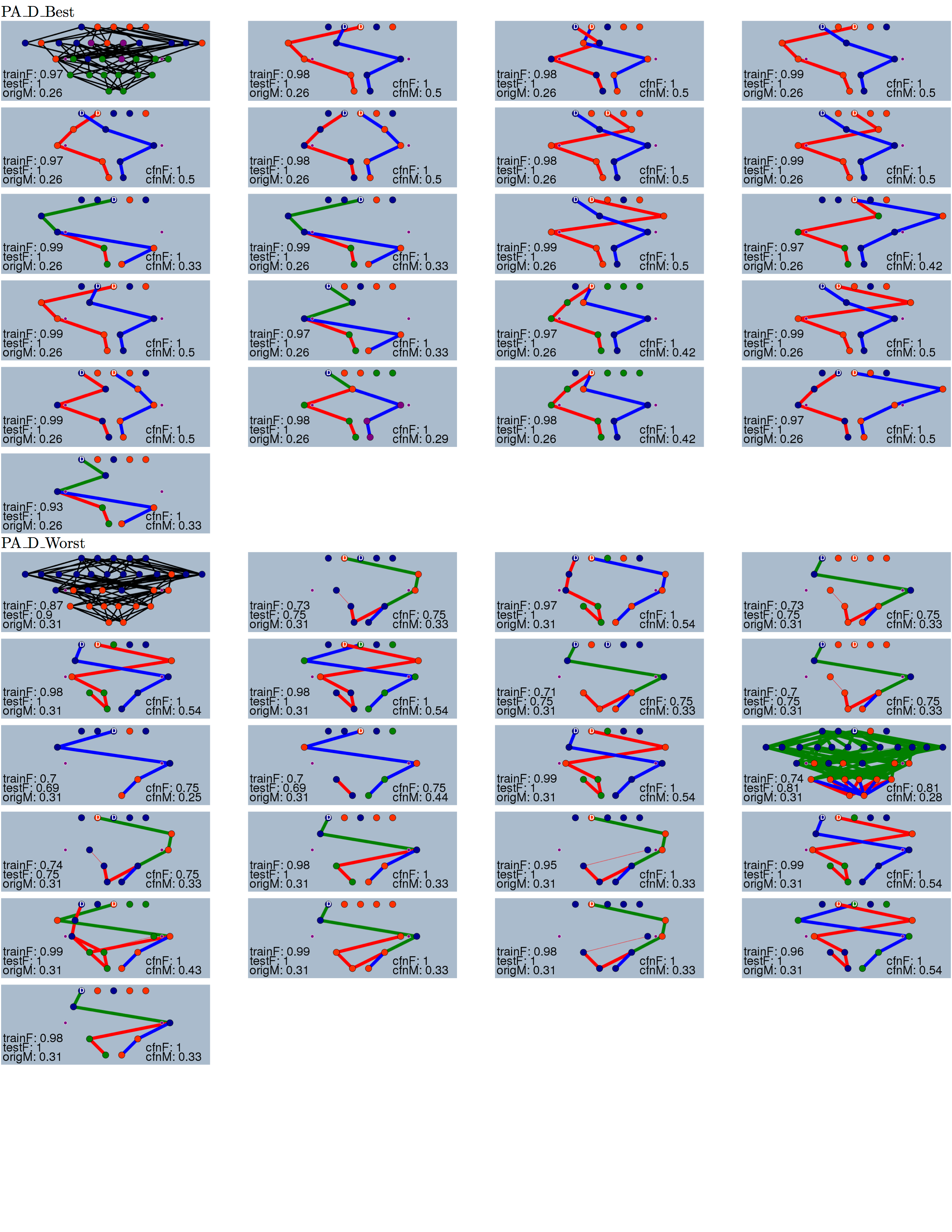

Supplement: S4 Fig — Each block contains the unsimplified version of the individual followed by 20 of its CFNs. (TIFF) [file pone.0187736.s005.tiff]

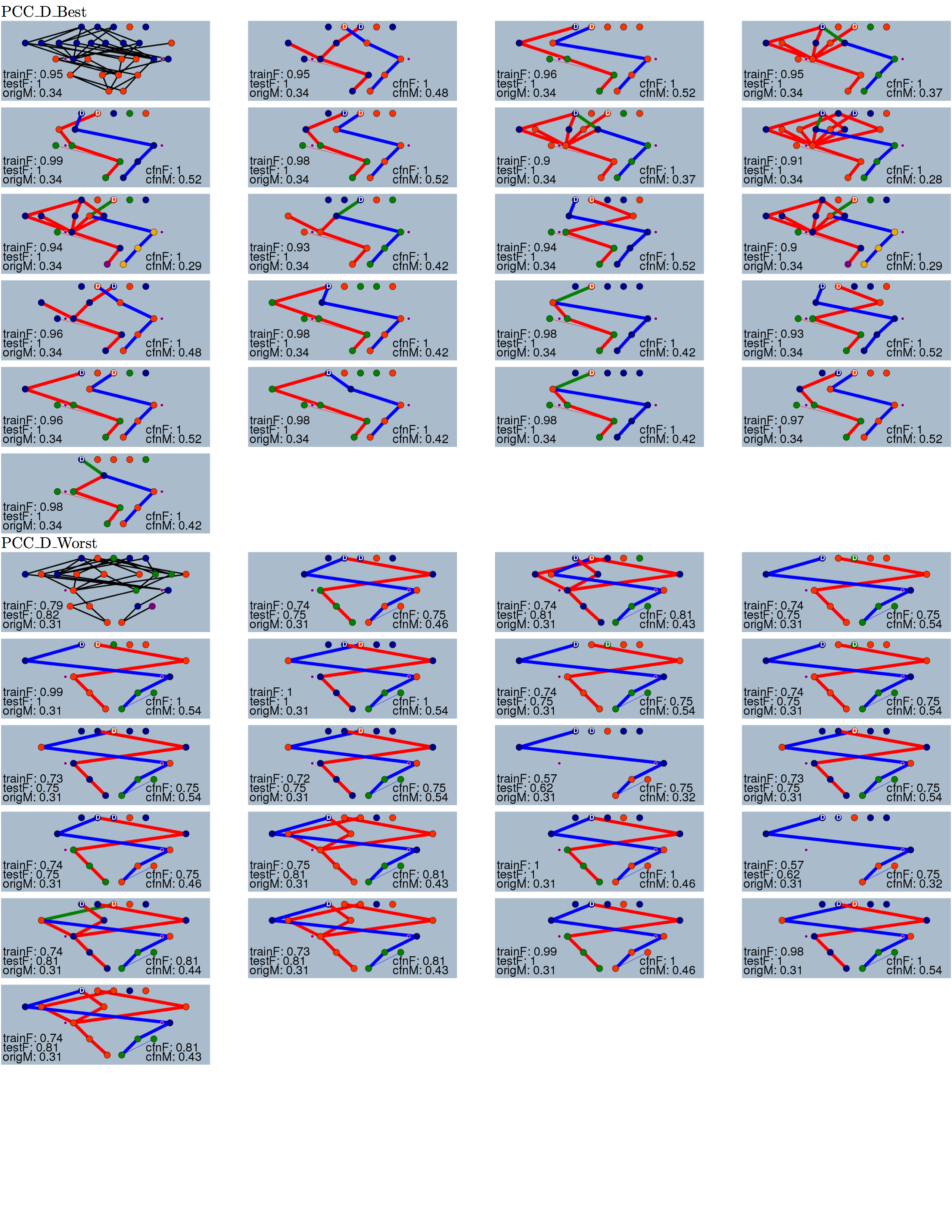

Supplement: S5 Fig — Each block contains the unsimplified version of the individual followed by 20 of its CFNs. (TIFF) [file pone.0187736.s006.tiff]

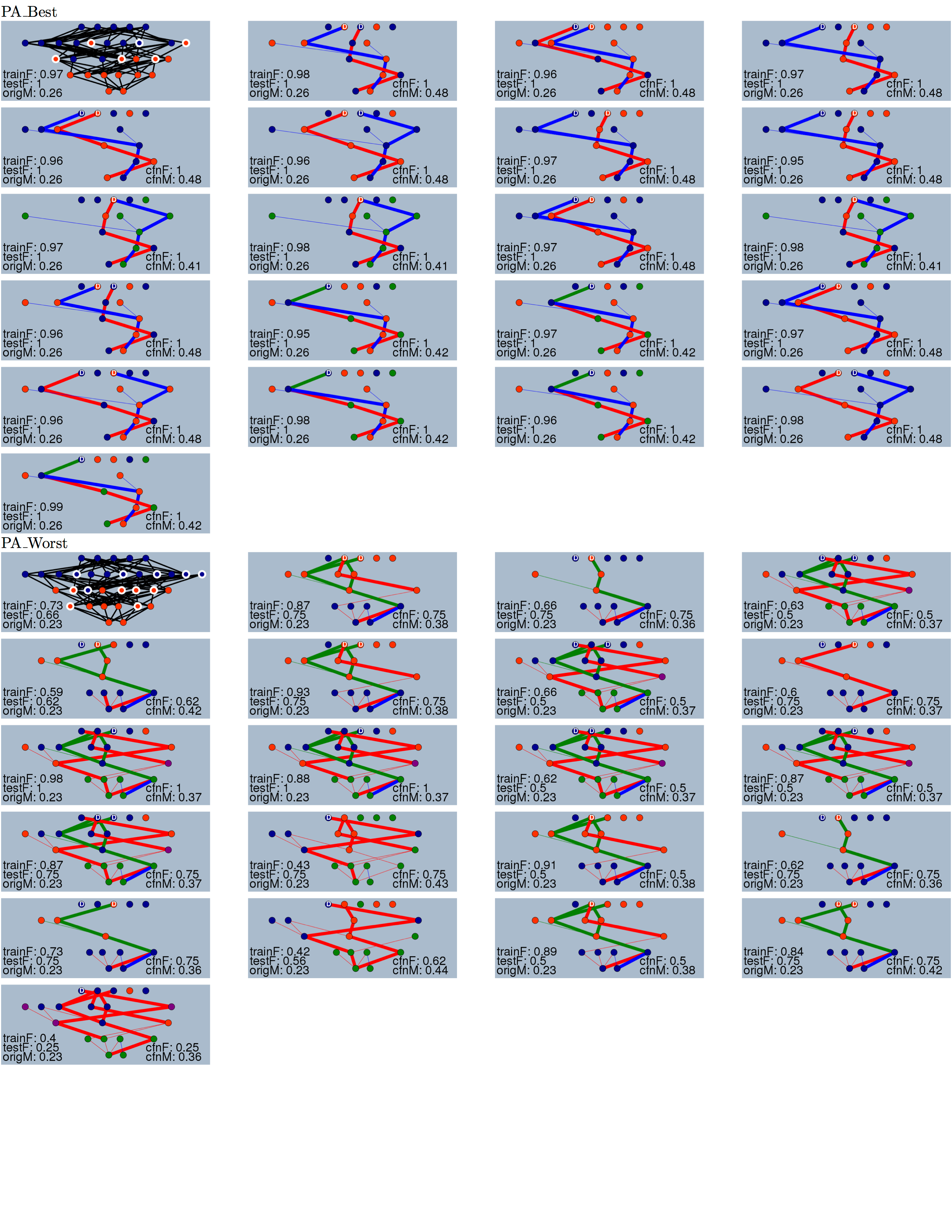

Supplement: S6 Fig — Each block contains the unsimplified version of the individual followed by 20 of its CFNs. (TIFF) [file pone.0187736.s007.tiff]

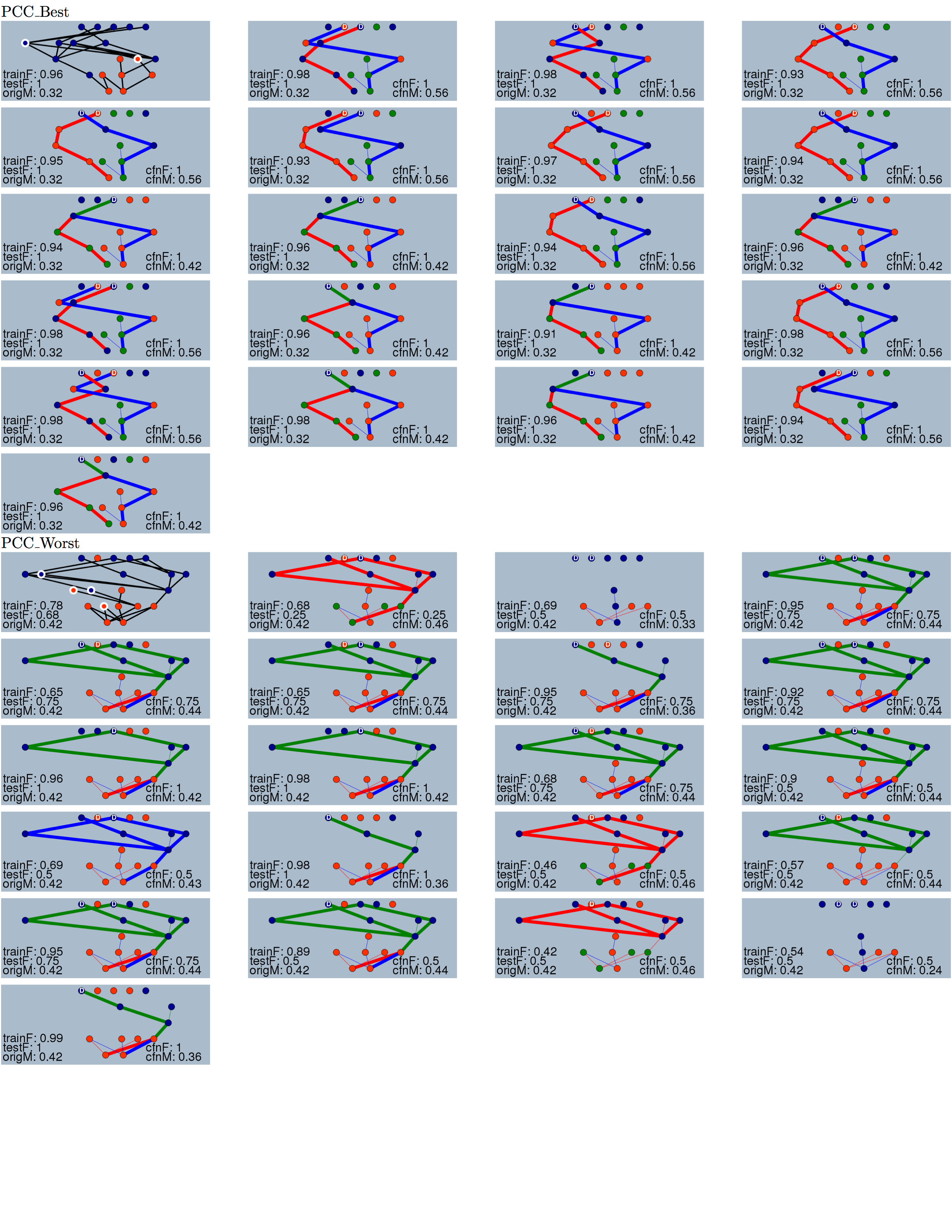

Supplement: S7 Fig — Each block contains the unsimplified version of the individual followed by 20 of its CFNs. (TIFF) [file pone.0187736.s008.tiff]
